# Improving the Catalytic Performance of Pectate Lyase Through Pectate Lyase/Cu_3_(PO_4_)_2_ Hybrid Nanoflowers as an Immobilized Enzyme

**DOI:** 10.3389/fbioe.2020.00280

**Published:** 2020-04-03

**Authors:** Pan Wu, Feifan Luo, Zhenghui Lu, Zhichun Zhan, Guimin Zhang

**Affiliations:** ^1^State Key Laboratory of Biocatalysis and Enzyme Engineering, Hubei Collaborative Innovation Center for Green Transformation of Bio-Resources, School of Life Sciences, Hubei University, Wuhan, China; ^2^Wuhan Sunhy Biology Co., Ltd., Wuhan, China

**Keywords:** pectate lyases, enzyme immobilization, hybrid nanoflowers, thermostability, textile industry

## Abstract

Pectate lyases (Pels) can be used in the textile industrial process for cotton scouring and ramie degumming, and its hydrolyzed products oligo galacturonic acid, are high-value added agricultural and health products. In our previous studies, an alkaline pectate lyase PEL168 mutant, PEL3, was obtained with improved specific activity and thermostability. Here, a facile and rapid method for preparing an immobilized PEL3-inorganic hybrid nanoflower was developed, as it could improve its biocatalytic performance. With 0.02 mg/mL (112.2 U/mL) PEL3 in PBS buffer, five different divalent ions, including Mn^2+^, Ca^2+^, Co^2+^, Zn^2+^, and Cu^2+^, were used as inorganic component. The results showed that PEL3/Cu_3_(PO_4_)_2_ hybrid nanoflowers presented the highest relative activity with 2.5-fold increase, compared to the free PEL3. X-ray diffraction analysis confirmed that the composition of PEL3/Cu_3_(PO_4_)_2_ hybrid nanoflowers were pectate lyase PEL3 and Cu_3_(PO_4_)_2_⋅5H_2_O. The optimum temperature and pH of PEL3/Cu_3_(PO_4_)_2_ hybrid nanoflowers were ascertained to be 55°C and pH 9.0, respectively, exhibiting subtle difference from the free PEL3. However, the PEL3/Cu_3_(PO_4_)_2_ hybrid nanoflowers maintained 33% residual activity after 24 h incubation at 55°C, while the free PEL3 completely lost its activity after 18 h incubation at 55°C. Furthermore, over 50% residual activity of the PEL3/Cu_3_(PO_4_)_2_ hybrid nanoflowers was remained, even after four times of repetitive utilization, demonstrating its promising stability for practical application.

## Introduction

Pectate lyases (Pels, EC 4.2.2.2) cleave α-1, 4-linked unesterified galacturonic acid motif of pectin by β-elimination, generating an unsaturated C4–C5 bond at the non-reducing end of the newly formed oligo galacturonic acid (OGA) (Sakai et al., 1993). Pels have potential application in textile industry, especially as ramie degumming and cotton scouring, which can replace conventional chemical process and produce high quality textiles with environment friendly (Zheng et al., 2001; Jayani et al., 2005). In addition, the hydrolyzate of pectin, OGA, has potential application in agriculture, especially used as potent plant growth promoter, as well as to improve anti-diseases ability in plants. OGA can be also used in health care industries, as an active ingredient of “functional foods” which are similar in appearance to conventional foods that are consumed as part of a normal diet and have physiological benefits to reduce the risk of chronic disease beyond basic nutritional functions (Clydesdale, 1997; Babbar et al., 2016). Due to its industrial applications, isolation and purification of Pels from wide-type strains such as *Bacillus* (Klug-Santner et al., 2006), *Erwinia* (Lojkowska et al., 1995), and *Aspergillus* (Dinu et al., 2007) were widely reported. Also, gene cloning and heterologous expression, molecular modification, and construction of high yield strain, etc., were studied and reported (Zhang et al., 2013; Kohli and Gupta, 2015; Liang et al., 2015; Wang et al., 2018).

Generally, Pels were active in alkaline condition, and additional Ca^2+^ are required for efficient enzymatic action. However, few natural Pels were found to be economically efficient for industrial applications because of their low activity or poor stability under the moderate temperature 40–70°C and alkaline pH 8–11, which was required processing conditions for industry (Zhang et al., 2013; Liang et al., 2015). Therefore, under the industrial processing conditions, Pels with higher activity and stability are still in demand. At present, directed evolution and rational design were the most successful strategies applied for engineering natural enzymes, and some successful examples of modified Pels have been reported to be compatible with the target industrial process (Liang et al., 2015; Wang et al., 2018). However, lacking of long-term operational stability and difficulty in reusability of Pels still hamper their use in industrial process. To overrule the above challenges, enzyme immobilization is one of the effective solutions (Ulf et al., 2009).

Enzyme immobilization has been considered as an efficient strategy to improve the specific activity and stability of enzyme, as it has the advantage of reusability, thereby reducing enzyme cost and high industrially feasible (Sheldon and Sander, 2013). The traditional methods of enzyme immobilization are binding, entrapping, and cross-linking enzyme to carriers (resins, polysaccharides, mesoporous silica, etc.) (Sheldon and Sander, 2013). Nevertheless, the disadvantages of these immobilization methods are obvious. For instance, binding force is too weak to release enzyme from carriers, and the activity was always decreased by conformational changes or accidently cross-linking the active sites of enzyme (Jesionowski et al., 2014). Recently, some novel strategies for enzymatic immobilization were developed based on new immobilized supporters such as nanomaterials, magnetic materials, and chemical modified supporters (Yang et al., 2016; Zucca et al., 2016; Liu et al., 2017). Among them, a facile and rapid preparation of protein–inorganic hybrid nanoflowers was first accidently discovered by Ge et al. (2012).

In protein–inorganic hybrid nanoflowers method, protein with metal ions in phosphate buffer saline (PBS) formed a flower-like nanoparticles using copper phosphate as the inorganic component in mild condition (25°C). Protein has a role of inducing crystal phosphate to form crystal nucleus to bury itself in the structural center of nanoflowers. This enzyme immobilization method has been used in catalase (Shi et al., 2014), bovine serum albumin (BSA) (Ge et al., 2012), lipase (Zhang et al., 2016b), horseradish peroxidase (Lin et al., 2014a), α-chymotrypsin (Yin et al., 2015), α-amylase (Wang et al., 2013), trypsin (Lin et al., 2014b), and papain (Zhang et al., 2016a), using different metal ions as the inorganic component. Most of the protein–inorganic hybrid nanoflowers showed improved activity and thermostability. As it was a recently developed technology, protein–inorganic hybrid nanoflower strategy has not been applied in Pels yet.

In our previous studies, a pectate lyase PEL168 from *Bacillus subtilis* 168 was cloned, characterized, and molecular modified (Zhang et al., 2013; Wang et al., 2018). A triple-site mutagenesis mutant K47D/V132F/R272W (PEL3) was obtained with specific activity of 5610 U/mg at 1 mM Ca^2+^, which showed the highest activity of reported alkaline Pels, and an extended T_50_ to 330 min at 50°C (Wang et al., 2018). In order to further improve the thermostability and reusability of PEL3, a facile and rapid method of preparing immobilized enzyme PEL3–inorganic hybrid nanoflowers was developed in this study. As the first report of preparing organic–inorganic hybrid nanoflowers of Pels, the thermostability of PEL3 inorganic hybrid nanoflowers was improved significantly, and it also could be reusable well.

## Materials and Methods

### Materials

*Escherichia coli* Rosetta (DE3)/pET28a-*pel3* for the expression of pectate lyase PEL3 was constructed in our previous work (Wang et al., 2018). Glycine with purity 98.5% and polygalacturonic acid (PGA) with purity 90% were purchased from Sigma-Aldrich (St. Louis, MO, United States). CuCl_2_, ZnSO_4_, MnSO_4_, CoCl_2_, CaCl_2_, KCl, NaCl, Na_2_HPO_4_, KH_2_PO_4_, Tris–HCl, and NaOH were purchased from Sinopharm Chemical Reagent Co., Ltd. All above chemicals were analytical grade. PBS buffer (10 mM) was prepared as follow: 4.00 g of NaCl, 0.10 g of KCl, 0.72 g of Na_2_HPO_4_, and 0.12 g of KH_2_PO_4_ were dissolved in 500 mL deionized water.

### Preparation of PEL3–Inorganic Hybrid Nanoflowers

For the synthesis of the protein–inorganic hybrid nanoflowers with improved catalytic performance, five different metal ions including Cu^2+^, Ca^2+^, Mn^2+^, Co^2+^, and Zn^2+^ were screened. First, we expressed and purified PEL3 according to our previously published method (Wang et al., 2018). Then, the purified protein PEL3 was diluted in PBS (pH 7.4) to get the protein solution of 0.02 mg/mL. Then, 16 μL of 1 M CuCl_2_, CaCl_2_, MnSO_4_, CoCl_2_, or ZnSO_4_ in milliQ water and 2 mL protein solution were gentle mixed, respectively, and followed by incubation at 25°C for 3 days. The purified PEL3 in PBS buffer without any metal ions mentioned above was incubated at 25°C for 3 days as a control. Finally, PEL3–inorganic hybrid nanoparticles were separated by centrifugation. The precipitates were washed thrice with PBS buffer, and dried by vacuum freeze-drying for further characterization. At the same time, the supernatant was collected to measure the protein concentration. The loading rate of PEL3 was calculated as “(1 - concentration of PEL3 in supernatant/initial concentration of PEL3) × 100%.” The effects of initial concentration of PEL3 on loading rate of PEL3, and morphology of the hybrid nanoflowers were also investigated. PEL3/Cu_3_(PO_4_)_2_ nanoparticles were produced when the concentration of PEL3 was augmented to 0.035, 0.05, or 0.07 mg/mL, respectively, while other conditions remained unchanged.

### Characterization of PEL3/Cu_3_(PO_4_)_2_ Hybrid Nanoflowers as Materials

The morphology of the nanoflowers were observed by Scanning Electron Microscope (SEM, JEOL JSM-7100F) at 10 kV. For SEM, the samples were sputtered coated with gold. Fourier Transform Infrared (FT-IR) spectra were acquired on a CY2000 FTIR spectrometer (Beijing Zhonghui Technology Co., Ltd). The measurements were performed using KBr pressed pellet method. Each sample was scanned at a range of 500–4000 cm^–1^ wavenumbers. Powder X-ray diffraction (XRD) patterns were recorded on a D8 Advance diffractometer (Bruker) with a monochromatized Cu Kα radiation source and a wavelength of 0.1542 nm.

### Enzymatic Characterization of PEL3/Cu_3_(PO_4_)_2_ Hybrid Nanoflowers

The activity of pectate lyase PEL3 was evaluated by measuring the level of galacturonic acid released in the enzymatic reactions with 0.2% (w/v) PGA as substrate, followed by A235 method (Wang et al., 2018). The effects of temperature on the enzymatic activities of free PEL3 and PEL3/Cu_3_(PO_4_)_2_ hybrid nanoflowers were measured in the temperature range of 20–70°C in glycine–NaOH buffer (pH 9.0). The effects of pH on the enzymatic activities were assayed in the glycine–NaOH buffers of pH ranging from 8.6 to 9.4 at 55°C.

Thermal stability of free PEL3 and PEL3/Cu_3_(PO_4_)_2_ hybrid nanoflowers were investigated by determining the residual activities after pre-incubation in glycine–NaOH buffer (pH 9.0) at 55°C with different time (0, 0.5, 1, 2, 4, 6, 12, 18, and 24 h). The residual activity (%) was the ratio between the activity of every sample and the maximum activity of the sample. The reusability of free PEL3 and PEL3/Cu_3_(PO_4_)_2_ hybrid nanoflowers was performed at 55°C in glycine–NaOH buffer (pH 9.0) with the method mentioned above. The same enzyme activity assay was used in three recycles. For every repetitive cycle, the hybrid nanoflowers were collected from the reaction solution and washed with PBS buffer for three times.

## Results

### Purification of PEL3 and Screening Metal Ions for Preparation of Pectate Lyase PEL3/Inorganic Hybrid Nanoflowers

**FIGURE 1:**
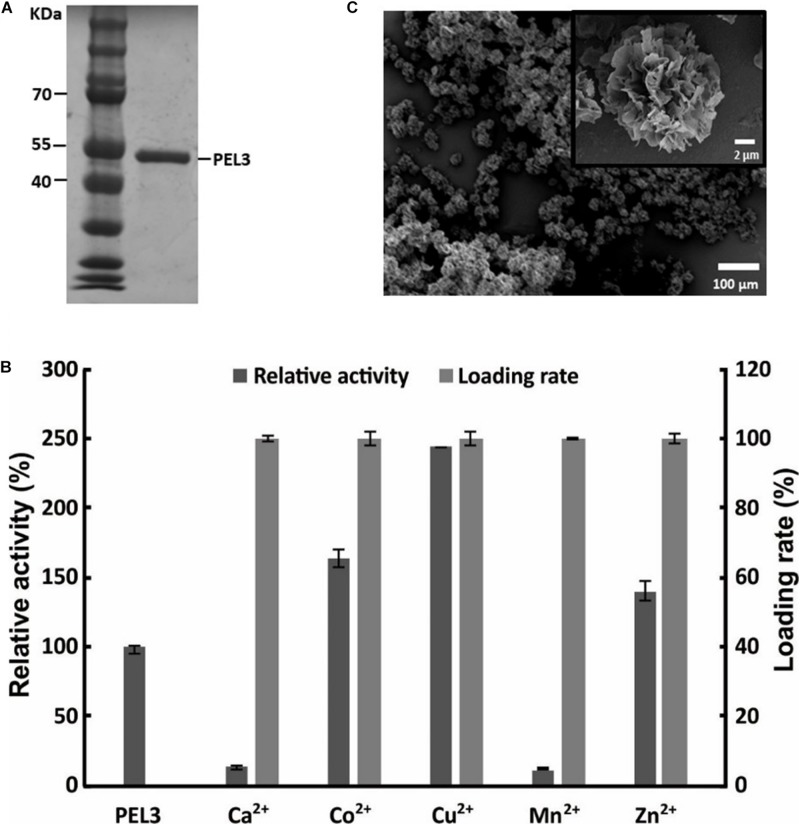
**(A)** SDS-PAGE analysis of purified pectate lyase PEL3. **(B)** Screening metal ions for preparation of PEL3/inorganic hybrid nanoflower. **(C)** SEM image of PEL3/Cu_3_(PO_4_)_2_ hybrid nanoflowers.

The pectate lyase PEL3 used for the preparation of hybrid nanoflower was purified and evaluated by SDS-PAGE, and the purified PEL3 appeared as a single band with a molecular mass of about 46 kDa (Figure 1A), consistent with the theoretically calculated value. Since the best protein–inorganic hybrid nanoflowers can be prepared using different metal ions as the inorganic part, five different metal ions including Cu^2+^, Ca^2+^, Mn^2+^, Zn^2+^, and Co^2+^ were screened. The results showed that the loading rate of PEL3 was nearly 100% with all metal ions tested in the synthesis of hybrid nanoflower, but the relative activity of free PEL3 incubated at 25°C for 3 days and PEL3/inorganic hybrid nanoflowers were different (Figure 1B). Among these PEL3–inorganic hybrid nanoparticles, PEL3/Cu_3_(PO_4_)_2_ hybrid nanoflowers presented the highest relative activity with a nearly 2.5-fold increase, when compared to the free PEL3 incubated at 25°C for 3 days. Comparably, the relative activity of PEL3/Co_3_(PO_4_)_2_ hybrid nanoparticles and PEL3/Zn_3_(PO_4_)_2_ hybrid nanoparticles were increased by 1.6-fold and 1.4-fold compared to the free PEL3, respectively. Surprisingly, the activity of free PEL3 was significantly improved by nearly fourfold in the presence of 1 mM Ca^2+^ (Wang et al., 2018), but the activity of PEL3/Ca_3_(PO_4_)_2_ hybrid nanoparticles was only remained 13% of free PEL3.

To identify the formation of regular hybrid nanoflowers, the precipitates of PEL3 and Cu_3_(PO_4_)_2_ were observed by SEM. The average particle size of the hybrid nanoflowers was about 20 μm. It showed that Cu^2+^ could promote the formation of PEL3/Cu_3_(PO_4_)_2_ hybrid nanoflowers with appearance of blooming flowers (Figure 1C).

### The Effects of Initial Concentration of PEL3 on Preparation of PEL3/Cu_3_(PO_4_)_2_ Hybrid Nanoflowers

**FIGURE 2:**
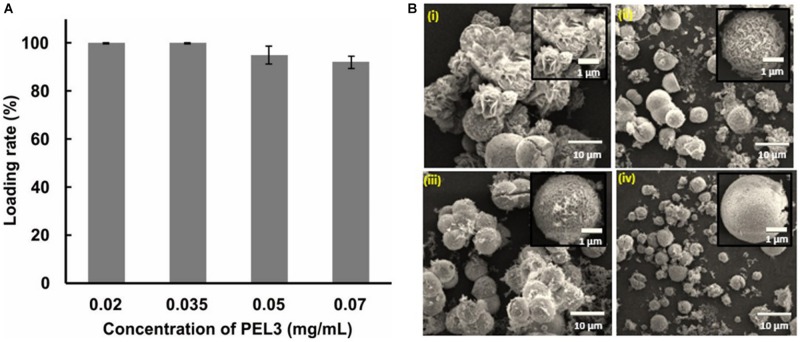
**(A)** Effects of initial concentration of PEL3 on loading rate. **(B)** Effects of initial concentration of PEL3 on morphology of Pel3/Cu_3_(PO_4_)_2_ hybrid nanoparticles. SEM image of PEL3/Cu_3_(PO_4_)_2_ hybrid nanoparticles with different concentration of PEL3 for preparation: **(i)** 0.02 mg/mL; **(ii)** 0.035 mg/mL; **(iii)** 0.05 mg/mL; and **(iv)** 0.07 mg/mL.

The morphology of protein–inorganic hybrid nanoparticles prominently has an influence on catalytic efficiency (Ge et al., 2012), and the concentration of protein affects the morphology (Wang et al., 2013). To estimate the effects of different concentration of pectate lyase PEL3 on the morphology of PEL3/Cu_3_(PO_4_)_2_ hybrid nanoflowers, different initial concentrations of PEL3 were used in preparation of hybrid nanoflowers. After increasing the concentration of PEL3 in the reaction solution from 0.02 to 0.07 mg/mL, it was observed that the loading rate of PEL3 by immobilization was only slightly decreased (Figure 2A). However, the morphology of the nanoparticles completely changed (Figure 2B). The particles showed gradual change from flower-like to spheroidicity-type with intermediate crack. When adding Cu^2+^ to 0.02 mg/mL PEL3 solution, the nanoparticle took present of flower blooming with obvious petals (Figure 2Bi). Increasing the concentration of PEL3 to 0.035 mg/mL caused that the morphology of nanoparticles changed to sphere with many small petals on the surface (Figure 2Bii), and continuing to increase the PEL3 concentration to 0.05 mg/mL led to more intensive petals (Figure 2Biii). Finally, when the PEL3 concentration was increased to 0.07 mg/mL, many small cracks emerged on the surface of spherical nanoparticles (Figure 2Biv). It was shown that the growth speed of thickness of petals was more from center to edge with the increasing concentration of PEL3. And 0.02 mg/mL was the optimum concentration of PEL3 for preparation of PEL3/Cu_3_(PO_4_)_2_ hybrid nanoflowers, as it could synthesize flower-like structure and also PEL3 could be utilized maximally. We also attempted to evaluate with lower concentration of PEL3, however, we could not accurately quantitate the PEL3 concentration lower than 0.02 mg/mL by Bradford or Nanodrop assay. In addition, the activity of these nanoparticles were not evaluated since several previous studies indicated that high surface area of the nanoflower particles facilitated mass-transfer, which contributed the enhanced activity of protein–inorganic hybrid nanoflowers (Ge et al., 2012; Wang et al., 2013). Therefore, our results indicated that the initial concentration of PEL3 had a significant effect on morphology of PEL3/Cu_3_(PO_4_)_2_ hybrid nanoflowers.

### Characterization of PEL3/Cu_3_(PO_4_)_2_ Hybrid Nanoflower as Materials

**FIGURE 3:**
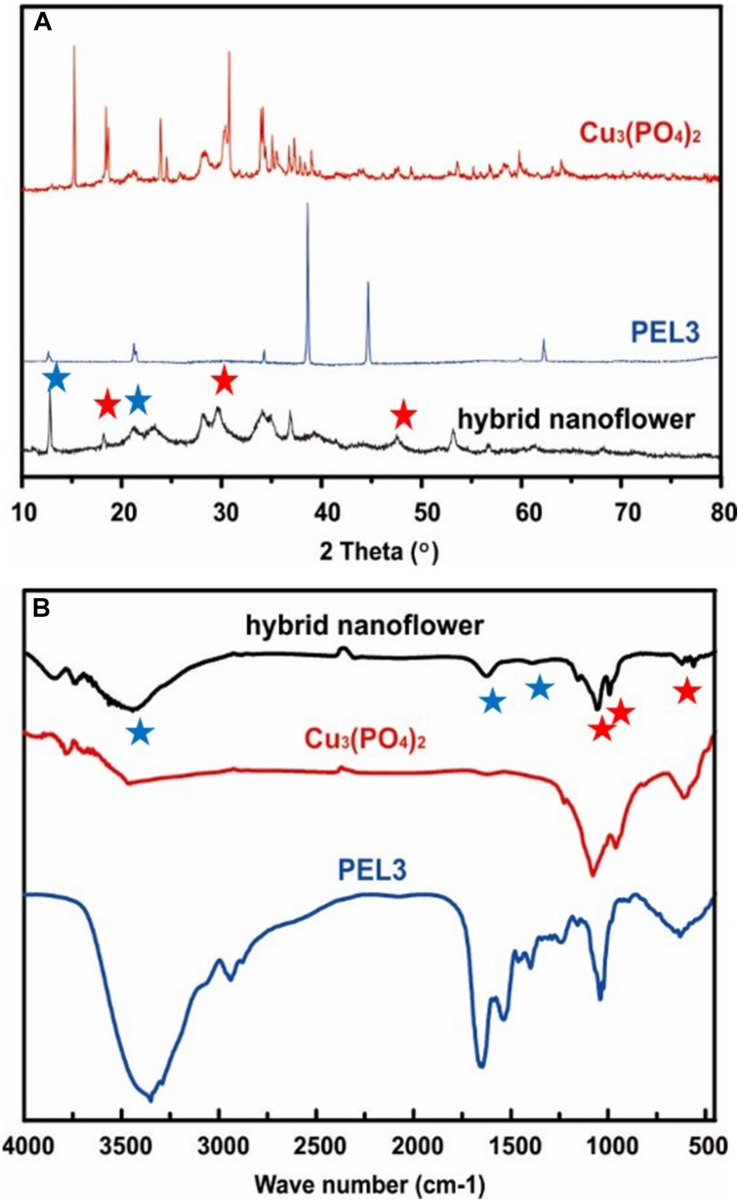
**(A)** X-ray diffraction patterns of pure PEL3, Cu_3_(PO_4_)_2_ nanoparticles and PEL3/Cu_3_(PO_4_)_2_ hybrid nanoflower. **(B)** FTIR spectra of pure PEL3, Cu_3_(PO_4_)_2_ nanoparticles and PEL3/Cu_3_(PO_4_)_2_ hybrid nanoflower. Red Pentagram are peaks attributed to Pel3, blue Pentagram are peaks attributed to Cu_3_(PO_4_)_2_.

The composition of PEL3/Cu_3_(PO_4_)_2_ hybrid nanoflower was characterized by X-ray powder diffraction (XRD) and Fourier transform infrared (FT-IR). The XRD spectrums of free PEL3, Cu_3_(PO_4_)_2_ nanoparticles and a typical PEL3/Cu_3_(PO_4_)_2_ hybrid nanoflowers were shown in Figure 3A. All the diffraction peaks in the XRD spectrum of Cu_3_(PO_4_)_2_ nanoparticles were in good agreement with Cu_3_(PO_4_)_2_⋅5H_2_O, which can be indexed by JCPDS (72–2356). The peaks at 18.79, 29.48, and 47.57° in spectrum of PEL3/Cu_3_(PO_4_)_2_ nanoflowers belonged to Cu_3_(PO_4_)_2_⋅5H_2_O nanoparticles, indicating that inorganic composition of the as-prepared hybrid nanoflowers was Cu_3_(PO_4_)_2_⋅5H_2_O. Besides, the peaks at 12.50° and 20.25° attributed to PEL3 were also observed in the spectrum of PEL3/Cu_3_(PO_4_)_2_ hybrid nanoflowers.

The FT-IR spectra of the samples from 500 to 4000 cm^–1^ are shown in Figure 3B. Distinctive absorption peaks of free PEL3 occurred at 1340–1430, 1670, and 3350 cm^–1^ for –CONH. The strong typical absorption peaks at 1058, 985, and 620 cm^–1^, which were observed in the spectrum of Cu_3_(PO_4_)_2_ nanoparticles, were attributed to P-O vibrations. It could be speculated that the characteristic absorption peaks of free PEL3 and Cu_3_(PO_4_)_2_ were maintained in spectrum of PEL3/Cu_3_(PO_4_)_2_ hybrid nanoflowers. These results proved that the nanoflowers were formed by pectate lyase PEL3 and Cu_3_(PO_4_)_2_⋅5H_2_O.

### Effects of Temperature and pH on the Activity of Free PEL3 and PEL3/Cu_3_(PO_4_)_2_ Hybrid Nanoflowers

**FIGURE 4:**
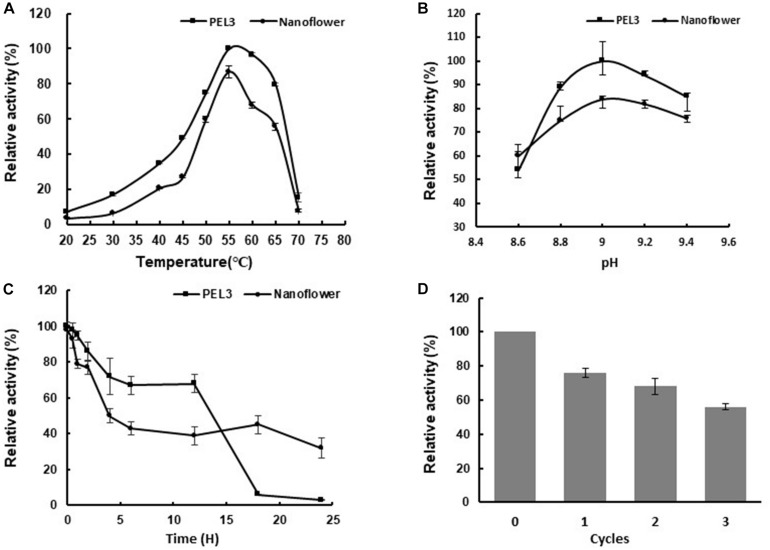
**(A)** Effects of temperature on the activity of free PEL3 and PEL3/Cu_3_(PO_4_)_2_ hybrid nanoflower. **(B)** Effects of pH on the activity of free PEL3 and PEL3/Cu_3_(PO_4_)_2_ hybrid nanoflower. **(C)** Effects of thermostability of free PEL3 and PEL3/Cu_3_(PO_4_)_2_ hybrid nanoflower. **(D)** Reusability of PEL3/Cu_3_(PO_4_)_2_ hybrid nanoflower. Each value of the assay was the arithmetic means of triplicate measurements.

Both the free PEL3 and PEL3/Cu_3_(PO_4_)_2_ hybrid nanoflowers presented the highest relative activity at 55°C (Figure 4A) and pH 9.0 (Figure 4B) toward PGA, while the relative activity of PEL3 was reduced about 20% after immobilization (Figures 4A,B). As PEL3 was an alkaline enzyme, the relative activity of PEL3 was retained above 50% from pH 8.6 to 9.4, even though it was immobilized as hybrid nanoflower. These results showed that the treatment of hybrid nanoflower did not affect the optimum temperature and pH of Pel, while the relative activity was slightly decreased.

### Reusability of PEL3/Cu_3_(PO_4_)_2_ Hybrid Nanoflowers

Enzyme immobilization could also benefit its reusability by improving the activity and stability to reduce the actual cost (Sheldon and Sander, 2013). The reusability of PEL3/Cu_3_(PO_4_)_2_ hybrid nanoflowers was investigated in this study. As shown in Figure 4D, the enzyme activity of the repetitive cycled hybrid nanoflowers diminished slowly as the cycle number increased. The hybrid nanoflowers can retain 56% of its initial activity after three times of consecutive reuse. It could be concluded that the designed immobilized PEL3 exhibited good operational stability and reusability, but it remained to be further improved.

## Discussion

Our studies demonstrated a facile and rapid strategy to achieve PEL3/Cu_3_(PO_4_)_2_ hybrid nanoflowers with improved thermostability and reusability. Pectate lyase PEL3 involved in our work was engineered, expressed, and purified by our previous works (Zhang et al., 2013; Wang et al., 2018). Comparably, the commercial enzymes such as BSA, laccase, lipase, α-amylase, etc., with uncertain purity were normally reported from different studies to generate hybrid nanoflowers (Ge et al., 2012; Wang et al., 2013; Zhang et al., 2016b). Although it is convenient to obtain large amount of enzymes by purchasing, commercial enzymes will be added some protective agents to extend their shelf life. It is uncertain if any of those ions or ingredients in commercial enzyme will influence the preparation of nanoparticles and catalytic performance. For PEL3, we can obtain a large amount of purified enzymes from the genetically engineered strain. This is the first report to present the research on protein–inorganic hybrid nanoflowers with knowledge of the purity and composition of the enzyme.

In this study, only 0.02 mg/mL PEL3 were used for preparing the PEL3/Cu_3_(PO_4_)_2_ hybrid nanoflowers, and the initial high concentration of PEL3 cannot form nanoparticle like flower-structure. The reasons for the above phenomenon was that PEL3 induced Cu_3_(PO_4_)_2_ to form a crystal nucleus, while PEL3 was embedded in the center of flower structure. The increase of PEL3 prompted the rapid and massive formation of PEL3/Cu^2+^, thus leading to a fast generation and growth of Cu_3_(PO_4_)_2_ nanoparticles with small particle *in situ*. In previous reports, most of the enzyme amount used for generation of nanoflowers particle was at least 0.2 mg/mL (Lee et al., 2015), except for α-lactalbumin/Cu_3_(PO_4_)_2_, laccase/Cu_3_(PO_4_)_2_, carbonic anhydrase/Cu_3_(PO_4_)_2_, and lipase/Cu_3_(PO_4_)_2_ hybrid nanoflowers (Ge et al., 2012). Comparably, the PEL3 used in preparation of PEL3/Cu_3_(PO_4_)_2_ hybrid nanoflowers was only 0.02 mg/mL, which was more than 10-fold less. In addition, the loading rate of PEL3 was approximately 100%, showing a complete utilization of PEL3 in the synthesis of PEL3/Cu_3_(PO_4_)_2_ hybrid nanoflowers. Moreover, PEL3 showed the highest activity among the reported alkaline Pels, less enzyme in the hybrid nanoflowers was enough to act on much substrate.

The preparation of protein–inorganic hybrid nanoflowers was first reported by using copper phosphate as the inorganic part (Ge et al., 2012). Although copper has been widely used for preparation of many nanoflowers, some reports also showed that copper phosphate was not the best choice in some cases. For example, the enzyme activity of lipase/Cu_3_(PO_4_)_2_ hybrid nanoflowers were evaluated to exhibit 5% decrease compared to free lipase, while lipase/Zn_3_(PO_4_)_2_ hybrid nanoflowers were increased 1.43-fold (Ge et al., 2012; Zhang et al., 2016b). Moreover, α-amylase/CaHPO_4_ hybrid nanoflowers (Wang et al., 2013), BSA/Mn_3_(PO_4_)_2_ hybrid nanoflowers (Zhang et al., 2015), and papain/Zn_3_(PO_4_)_2_ hybrid nanoflowers (Zhang et al., 2016a) were exhibited excellent catalytic performance. Besides that, cobalt ion has not been used in preparation for protein–inorganic nanoflower in the past, but it was always used in the preparation for immobilization carriers (Khan et al., 2017). Thus, in this study, five different ions including cobalt ion were screened for preparation of PEL3-inorganic hybrid nanoflowers. The results showed that copper phosphate was the optimum inorganic component of PEL3-inorganic hybrid nanoparticles and PEL3/Co_3_(PO_4_)_2_ hybrid nanoparticles presented 1.6-fold increased activity with almost 100% loading rate (Figure 1B). However, the activity of PEL3/Cu_3_(PO_4_)_2_ hybrid nanoflowers was decreased about 20% comparing to free PEL3 (Figures 4A,B), similar to the results of lipase/Cu_3_(PO_4_)_2_ hybrid nanoflowers (Ge et al., 2012). This indicates that metal ions sometimes can cause the denaturation of enzyme and thus diminish enzymatic activity during the self-assembly process (Wu et al., 2015).

As well as we know, most Pels are Ca^2+^-dependent metalloenzyme (Sharma et al., 2013). In our previous studies, both 1 and 5 mM Ca^2+^ improved 400% activity of PEL3 (Wang et al., 2018). Theoretically, adding Ca^2+^ in the preparation of enzyme/calcium phosphonate hybrid nanoparticles can promote the activity of Ca^2+^-dependent enzyme. It demonstrates the allosteric effect occurred when interacted with Ca^2+^ in aqueous solutions. According to this principle, Wang et al. (2013) designed and synthesized the α-amylase/CaHPO_4_ nanoflower, which improved the activity of α-amylase significantly. However, PEL3/calcium phosphonate hybrid nanoparticles almost lost the activity (Figure 1B). The reason of this phenomenon may be that the allosteric effect does not happen during PEL3/calcium phosphonate hybrid nanoparticles preparation, and the high concentration of calcium binds to calcium binding sites to inhibit its activity.

Pectate lyases PEL168 was produced from probiotic bacteria *B. subtilis*, which is safe for application in food, feed and textile industry (Sharma et al., 2013; Kohli and Gupta, 2015). Therefore, it has been identified and expressed in *E. coli* BL21 (DE3) for characterization and analysis of protein structure (Arefeh et al., 2010). In order to suit for industrial application of pectate lyases PEL168, Zhang et al. (2013) expressed PEL168 in *Pichia pastoris*, and the recombinant protein was glycosylated with removing pectin from ramie efficiently, which is an ideal candidate for further optimization and engineering for bio-degumming. This enzyme was also expressed in *E. coli* with high catalytic efficiency through fusion of amphipathic peptide strategy (Zhang et al., 2013). In addition, Wang et al. (2018) engineered PEL168 to obtain the benefit mutant PEL3 with increased specific activity and thermostability. The bioscouring assay exhibited that PEL3 can obviously improve the wettability and softness of fabrics, suggesting its potential application in textile industry (Wang et al., 2018). The first case for enzyme immobilization of pectate lyases (PGL) from *B. subtilis* was reported by Ran et al. (2017), PGL was successfully immobilized on the surface of polyhydroxyalkanoate (PHA) nanogranules by fusing PGL to the N-terminal of PHA synthase from *Ralstonia eutropha* via a designed linker. The activity of PGL–PHA compared to the free PGL was decreased almost 15%, and immobilization process did not affect the optimal pH and temperature of the free Pels, which was also observed with PEL3/Cu_3_(PO_4_)_2_ hybrid nanoflowers. However, PGL–PHA only retained less than 20% of its initial activity after incubation at 55°C for 12 h, while PEL3/Cu_3_(PO_4_)_2_ hybrid nanoflowers retained almost 40% of its initial activity after incubation at 55°C for 24 h. After re-use for three cycles, PGL–PHA could hold above 70% of its initial activity, but PEL3/Cu_3_(PO_4_)_2_ hybrid nanoflowers only retained around 56% of its initial activity. It may be due to PEL3/Cu_3_(PO_4_)_2_ hybrid nanoflowers higher specific activity, only small amount was used for activity assay. After each reaction, PEL3/Cu_3_(PO_4_)_2_ hybrid nanoflowers were recovered through centrifugation, which caused a magnificent loss especially when the pellet is very small. Although the operational stability of PEL3/Cu_3_(PO_4_)_2_ hybrid nanoflowers remains to be improved. It assumed that the actual activity loss should be less than 44% after re-use for three cycles. Therefore, owing to its highest specific activity in the reported alkaline Pels (Wang et al., 2018), PEL3 immobilized as hybrid nanoflowers with good thermostability and reusability are potential for industrial applications.

## Conclusion

In conclusion, the resultant hybrid nanoflower formed by PEL3 and copper phosphate exhibited excellent thermostability and good reusability compared with free pectate lyase PEL3. Therefore, the hybrid nanoflower materials as catalysts have great potential in industrial applications. It is also the first time to report organic–inorganic hybrid nanoflowers of Pels.

## Data Availability Statement

All datasets generated for this study are included in the article/supplementary material.

## Author Contributions

PW and FL performed the experiments. PW and GZ designed the experiments and wrote the manuscript. ZL and ZZ were involved in analysis and interpretation of experimental data. GZ conceived the idea and supervised the whole research. All authors read and approved the final manuscript.

## Conflict of Interest

PW and ZZ were employed by the company Wuhan Sunhy Biology Co., Ltd., Wuhan, China. The remaining authors declare that the research was conducted in the absence of any commercial or financial relationships that could be construed as a potential conflict of interest.
